# Supporting dataset for elemental traits of plant-invertebrate food web components of oilseed rape fields

**DOI:** 10.1016/j.dib.2019.104470

**Published:** 2019-09-05

**Authors:** Grzegorz Orłowski, Jerzy Karg, Piotr Kamiński, Jędrzej Baszyński, Małgorzata Szady-Grad, Krzysztof Ziomek, Jacek J. Klawe

**Affiliations:** a Institute of Agricultural and Forest Environment, Polish Academy of Sciences, Bukowska 19, 60-809, Poznań, Poland; b Department of Nature Conservation, Faculty of Biological Sciences, University of Zielona Góra, Prof. Z. Szafrana 1, 65-516, Zielona Góra, Poland; c Department of Medical Biology and Biochemistry, Collegium Medicum in Bydgoszcz, Nicolaus Copernicus University, M. Skłodowska-Curie 9, 85-094, Bydgoszcz, Poland; d Department of Biotechnology, Faculty of Biological Sciences, University of Zielona Góra, Prof. Z. Szafran St. 1, 65-516, Zielona Góra, Poland; e Department of Hygiene, Epidemiology and Ergonomics, Collegium Medicum in Bydgoszcz, Department of Hygiene and Epidemiology, M. Skłodowska-Curie 9, 85-094, Bydgoszcz, Poland

**Keywords:** Trace elements, Bioenergy crops, *Brassica napus*, Food web, Insects, Field margins

## Abstract

This dataset is provided in support of the paper "Edge effect imprint on elemental traits of plant-invertebrate food web components of oilseed rape fields" (Orłowski et al., 2019). Supplementary data are given on the following: (1) the full taxonomic list of invertebrates (*n* = 12 916) classified into food guilds and functional groups, which were sampled in 34 oilseed rape fields in SW Poland in spring 2015; (2) concentrations of 12 chemical elements measured in invertebrates; (3) the relationships between abundance and percentage (%) in the community of major invertebrate groups, and habitat variables; (4) the statistical tests comparing the concentrations of chemical elements between the different groupings of organisms; (5) the relationships between the elemental traits of oilseed rape plant samples and major functional invertebrate groupings or main taxonomic insect groups, and the habitat variables of oilseed rape fields.

**Table:** Specifications Table

Subject area	Ecology, Biological Sciences, Biogeochemistry, Agroecology
More specific subject area	Biogeochemistry of invertebrates
Type of data	Tables, figures
How data was acquired	Through field work and laboratory work
Data format	Raw, filtered and analysed
Experimental factors	Investigation of chemical composition of insects and plant tissues, and variability in land-cover.
Experimental features	Quantification of the abundance of invertebrates and measurements of the elemental composition (K, Na, Ca, Mg, Cu, Zn, Fe, Mn, As, Cd, Co and Pb) of 15 different organisms within the plant-invertebrate food web: plant – oilseed rape pests/herbivores – pollinators = wild bees – saprovores – predators – parasitoids. These were then related to the individual field edge habitat features (including typically anthropogenic ones like dirt and tarred roads) measured within a 100 m radius around the invertebrate sampling sites.
Data source location	The dataset presented in this data paper were gathered in spring 2015 on 35 winter oilseed rape fields (average area 22.46 ha; range 0.82–159.22 ha) in the agricultural landscape around the village of Turew, Wielkopolska province, south-west Poland.
Data accessibility Related research article	The data are given in this article G. Orłowski, J. Karg, P. Kamiński, J. Baszyński, M. Szady-Grad, K. Ziomek, J. Klawe, Edge effect imprint on elemental traits of plant-invertebrate food web components of oilseed rape fields. Sci. Tot. Environ. 687 (2019) 1285–1294. https://doi.org/10.1016/j.scitotenv.2019.06.022

**Table:** 

**Value of the data** • The data on elemental traits of organisms relate to the individual field edge habitat features (including typically anthropogenic ones like dirt and tarred roads) measured within a 100 m radius around the invertebrate sampling sites. • The data in this article demonstrate that the elemental traits of the plant-invertebrate food web components in oilseed rape crops varied owing to the habitat specificity determined at the relatively small spatial scale of an individual field, and that the elemental traits of these organisms differed from both an inter- and an intra-guild perspective. • These data may be useful for explaining the sources of variation in both the quality of agricultural products (including food for human consumption) and the dietary flow of essential macronutrients and non-essential trace elements within plant-invertebrate food webs in agroecosystems.

## 1 Data

**Fig. 1:**
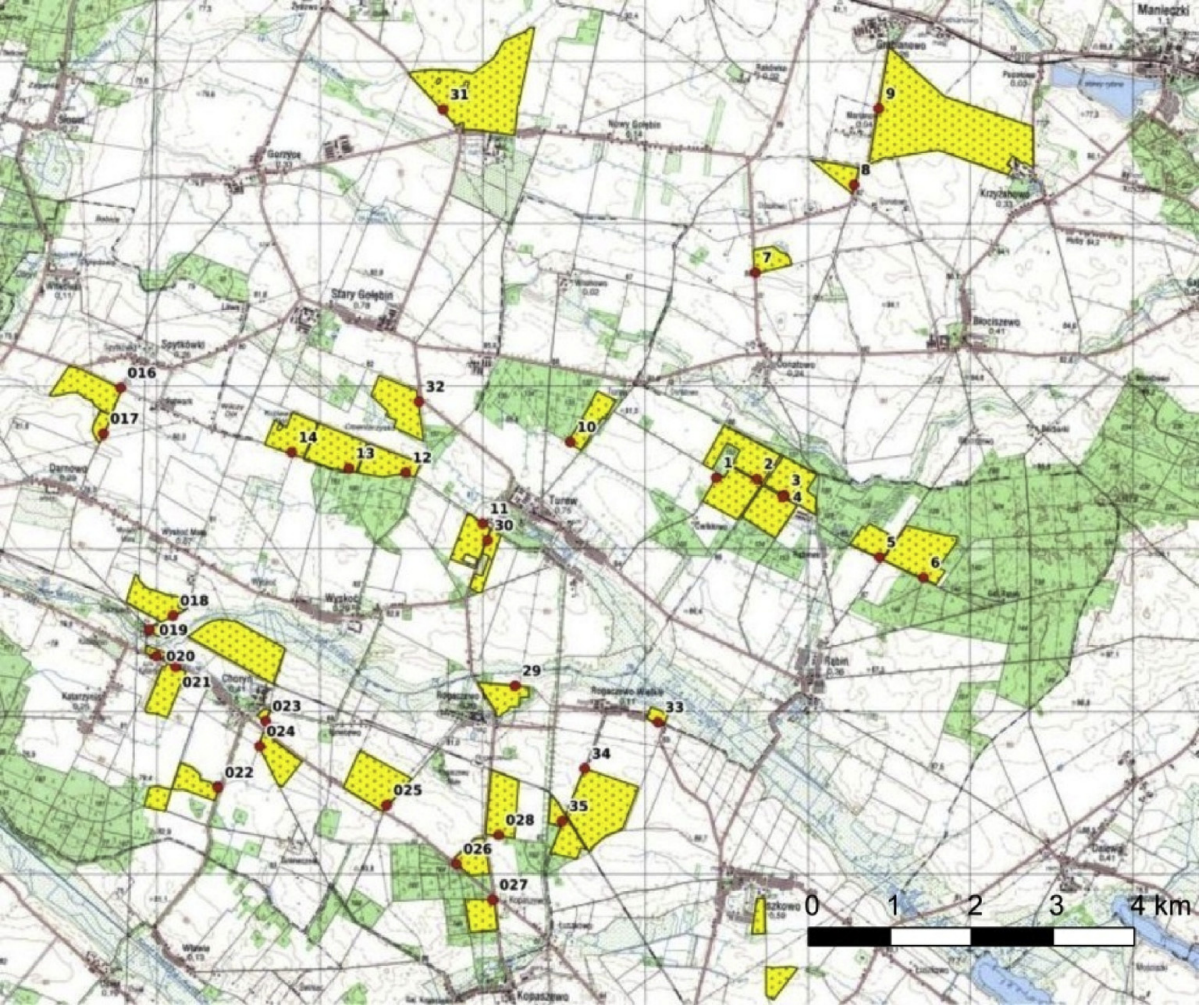
Distribution of the 34 oilseed rape fields with the exact positions of the Moericke traps (red dots), where invertebrates were sampled in spring 2015.

**Fig. 2:**
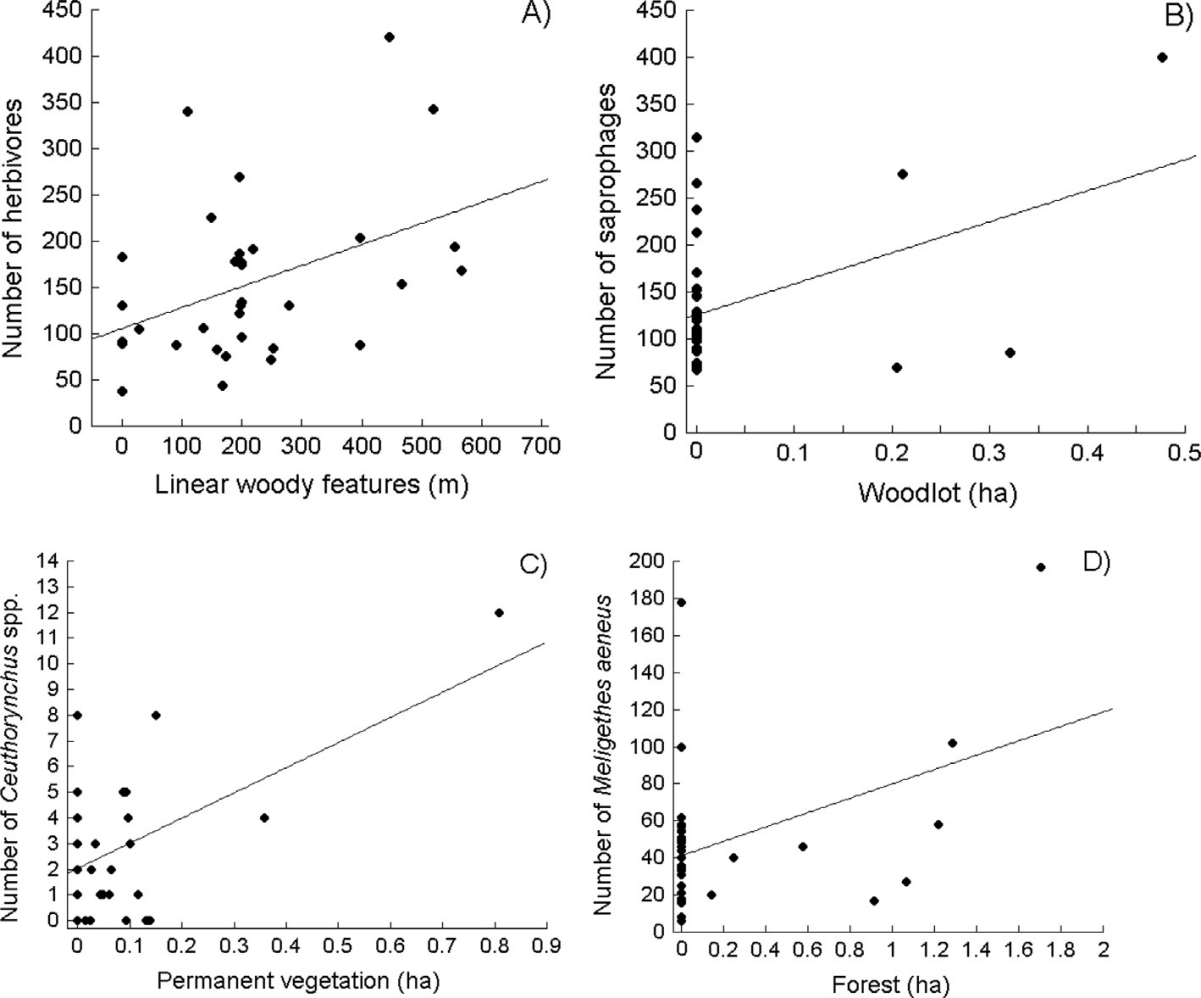
Examples of statistically significant relationships between the number of major invertebrate/insect groups sampled and the habitat variables of 34 oilseed rape fields; woodlot = wood.

The data presented here (Fig. 1, Fig. 2; Table 1, Table 2, Table 3, Table 4, Table 5, Table 6, Table 7) constitute the basis for the article by Orłowski et al. [1]. The dataset provides detailed information on: (1) the full taxonomic list of invertebrates (*n* = 12 916) classified into food guilds and functional groups (Annex 1 in Ref [1]; Table 4), which were sampled in 34 oilseed rape fields in SW Poland in spring 2015 (Fig. 1; Table 1, Table 2); (2) concentrations of 12 chemical elements measured in invertebrates (Table 3); (3) the relationships between abundance and percentage (%) in the community of major invertebrate groups, and habitat variables (Table 5); (4) the statistical tests comparing the concentrations of chemical elements between the different groupings of organisms (Table 6); (5) the relationships between the elemental traits of oilseed rape plant samples and major functional invertebrate groupings or main taxonomic insect groups, and the habitat variables of oilseed rape fields (Table 7).

**Table 1:** Basic descriptive statistics of 10 habitat variables (= landscape/land-cover features) measured within a 100 m radius (= 3.1384 ha) around 34 invertebrate sampling points in 34 oilseed rape fields between 0.82 and 159.22 ha in area; tarred road = asphalt road.

Landscape/land-cover feature (unit)	Average	−95% C.I.	+95 C.I.	Min.	Max
Arable land (ha)	2.46	2.27	2.65	0.93	2.92
Permanent vegetation: grassland, road verges (ha)	0.08	0.03	0.13	0	0.81
Linear woody features (m)	209.85	152.86	266.84	0	566.41
Linear woody features (ha)	0.181	0.120	0.241	0	0.946
Length of dirt road (m)	111.69	76.41	146.97	0	361.41
Coverage of dirt road (ha)					
Length of tarred (paved) road (m)	115.80	74.89	156.71	0	379.72
Coverage of tarred (paved) road (ha)					
Wood/mid-field copses (ha)	0.04	0.00	0.07	0	0.48
Forest (ha)	0.21	0.05	0.37	0	1.70
Wooded area (wood + forest + recently planted wood/forest) (ha)	0.27	0.10	0.45	0	1.70

**Table 2:** Component values and factor loadings of the Principal Component Analysis (PCA) of nine landscape/land-cover features measured within a 100 m radius around 34 invertebrate sampling points in 34 oilseed rape fields in SW Poland (see Table 1); factor rotation: varimax normalised; the figures in bold indicate the variable for which each factor exhibited the greatest variability.

Habitat variable	Axis (conventional description)			
	PC1 (Roads)	PC2 (Arable + woods)	PC3 (Hedge)	PC4 (Field area)
Field area (ha)	0.131	0.130	−0.133	**0.883**
Arable land (ha)	0.166	**−0.880**	0.051	0.319
Permanent vegetation (ha)	−0.354	−0.195	−0.489	−0.292
Linear woody features (ha)	0.196	−0.094	**0.876**	−0.135
Linear woody features (m)	−0.035	−0.119	**0.921**	−0.041
Dirt road (ha)	**0.905**	0.165	0.092	0.113
Dirt road (m)	**0.903**	0.120	0.208	0.023
Tarred road (ha)	**−0.904**	0.160	0.011	0.024
Tarred road (m)	**−0.942**	0.071	0.015	−0.012
Wood/mid-field copses (ha)	0.274	0.177	−0.381	−0.407
Forest (ha)	0.059	**0.901**	−0.022	0.288
Wooded area (ha)	0.121	**0.959**	−0.125	0.106
Eigenvalues	3.642	2.687	2.088	1.260
Variation explained	0.303	0.224	0.174	0.105

Note: The explanatory power of the above PCA derived variables as regards the abundance of the major invertebrate groups is poor: only PC2 (Arable + woods) was positively correlated with the numbers of *Meligethes aeneus* and oilseed rape pests, while %oilseed rape pests (Pearson *r* = 0.358, 0.362 and 0.359, *P* ≤ 0.048); and PC3 (Hedge) was negatively correlated with %saprovores (*r* = −0.396, *P* = 0.028).

**Table 3:** Concentrations of 12 chemical elements measured in 14 invertebrate/insect taxa/species sampled by sweep netting in oilseed rape fields in SW Poland in spring 2015.

Sample No.	Taxa/species (functional group; sampling date)	K	Na	Ca	Mg	Cu	Zn	Fe	Mn	As	Cd	Co	Pb
1	*Meligethes aeneus* (oilseed rape pest; 15.V.15)	150.4	807.8	296.1	176.3	77.93	0.62	381.4	2.85	0.05	2.48	0.13	0.77
2	*Ceutorhynchus assimilis* (oilseed rape pest; 15.V.15)	150.4	767.1	284.0	185.1	71.69	4.68	135.1	1.28	0.41	1.71	0.26	1.76
3	*Prosternon tesselatum* (herbivore; 15.V.15)	150.4	809.6	287.1	187.6	86.15	5.00	402.9	3.18	0.05	5.05	0.20	1.70
4	*Dolycoris baccarum* (herbivore; 20.V.15)	28.2	763.1	261.7	164.2	77.56	26.38	363.4	8.34	0.11	1.83	0.40	1.85
5	*Oulema melanopus* (herbivore; 15.V.15)	26.86	732.2	263.6	158.9	38.20	7.68	487.1	9.69	0.13	1.74	0.25	1.87
6	*Phyllobius* sp. (herbivore; 15.V.15)	15.31	807.2	283.1	161.0	102.8	4.52	120.1	0.59	0.03	1.69	0.22	1.91
7	*Nabis ferus* (herbivore; 20.V.15)	21.83	777.4	339.2	163.3	124.8	6.85	225.9	0.55	0.04	2.91	0.21	2.13
8	*Scatophaga stercoraria* (saprovore; 20.V.15)	14.45	836.5	302.7	141.9	106.9	5.91	200.0	0.52	0.02	1.94	0.23	2.18
9	*Bibio hortulanus* (saprovore; 20.V.15)	9.82	805.5	287.7	152.5	107.8	8.02	238.3	0.36	0.01	1.56	0.21	2.19
10	Muscidae, Bibionidae, Calliphoridae (saprovore; 15.V.15)	5.81	799.5	294.1	158.6	108.6	6.76	232.4	0.19	0.09	1.71	0.25	2.19
11	Araneae (predator; 15.V.15)	19.65	827.7	238.8	187.6	31.32	24.39	407.5	5.60	0.07	2.05	0.27	2.00
12	*Cantharis fusca* (predator; 20.V.15)	138.3	851.2	269.3	173.0	17.89	9.48	301.7	0.53	0.01	1.57	0.23	2.09
13	Ichneumonidae (parasitoid; 15.V.15)	17.08	773.6	324.3	148.6	120.5	4.10	197.2	0.64	0.02	1.77	0.27	2.20
14	*Tersilochus heterocerus* parasitoid of *Meligethes aeneus* (parasitoid; 15.V.15)	90.54	723.0	290.0	160.5	107.6	10.62	276.9	0.48	0.03	1.62	0.15	2.31

**Table 4:** The full taxonomic list of invertebrates (*n* = 12 916), classified into food guilds and functional groups, which were sampled in 34 oilseed rape fields in SW Poland in spring 2015. In each field, invertebrates were sampled using two yellow Moericke traps on six sampling days: 13 April, 16 April, 20 April, 23 April, 27 April and 5 May 2015.

Order	Family/taxa	Food guild		Food type	Functional group	#Field																																		Total
						1	2	3	4	5	6	7	8	9	10	11	12	13	14	16	17	18	19	20	21	22	23	24	25	26	27	28	29	30	31	32	33	34	35	
	Araneae	predator		insects	predator	2	3	2		1		6	3	1		1	2		2	23	3	6	2			3		2		5	3		3	2	1	5	2	1	1	85
	Thysanoptera	herbivorous		grass	herbivorous		1	2						1				1			1				1	1			1		2		1		1					13
	Aphididae	herbivorous		plants	herbivorous					1			1		2				1			1				1				11	1					2		2		23
DIPTERA																																								
	Sciaridae	saprovorous		organic matter	saprovorous	116	47	46	32	31	23	43	53	116	77	47	17	48	52	27	27	44	18	20	47	50	53	191	47	45	32	250	37	21	41	24	19	33	27	1801
	Chironomidae	saprovorous		organic matter	saprovorous	89	9	14		15	10	66	40	66	12	22	14	21	22	6	25	19	12	17	8	6	21	72	15	2		85	14	7	3	3	6	21	17	759
	Cecidomyiidae	herbivorous		plants	herbivorous	3	1	1		4	2	1	9	24	5		2		2			6	1			4	2	21	2			8	4		1		2	6	2	113
	Phoridae	saprovorous		organic matter	saprovorous	6	8	11	3	1	5	1	4	4	3	3	7	4	2	4	2	1	1	5	5	4	3	3	1	1	7	3	3	3	1	1	3			113
	Cypselidae	saprovorous		organic matter	saprovorous	22	8	15	3	21	9	9	34	9	36	11	5	13	8	5	13	10	23	9	11	9	15	8	10	11	7	15	10	2	8	5	12	5	4	395
	Crossopalpus	predator		insects	predator	2	1	3	1	3	1	2	1		2		2	2			1	1			1		1													24
	Empididae	predator		insects	predator	2		1	2		4	1	2				1		1	1									1									2		18
	Drosophilidae	saprovorous		organic matter	saprovorous	3	6	5	1	2	6		11	17	4	18	1	6	3	4	3	5	3	3	2	6	6	3	1		6	6	14	7	17	1	2		5	177
	Muscidae	herbivorous		plants	herbivorous	49	165	106	172	151	235	137	83	143	57	101	63	111	116	50	171	87	14	28	63	167	149	191	406	55	153	56	148	60	118	323	62	178	80	4248
	Fungivoridae	saprovorous		organic matter	saprovorous	1			1	1	1		1					1			1	2	1				1	1		1	5	1			1		1	2	1	24
	Chloropidae	herbivorous		plants	herbivorous	3		2	7		1	2		2	2		1	2	1	1			9	2	1	3	2	12	2	1		4	3	1	1			4		69
	Bibionidae	detrivorous		organic matter	saprovorous	2	35	8	19	27	3	89	6	28	40	9	15	11	17	1	2	9		6	8	8	25	15	57	7	9	4	61	15	42	21	55	18	20	692
	Scatophagidae	coprophagous		organic matter	saprovorous	1				1	4	12	4	3	4			2			5	1	1	2	1	4	8	3	2	3			2		2		4			69
	Musidoridae	saprovorous		organic matter	saprovorous	1				1		1		1	1			2	1											1							1			10
	Syrphidae	predator		insects	predator		5			3	48		1	1	3		1	4					2			3	1		1	5	2						1			81
	Anthomyiidae	herbivorous		plants	herbivorous					2	25	1					1	2		1	2		2		6		8	99	1		1	7		23			7	18	5	211
	Sepsidae	saprovorous		organic matter	saprovorous					1						1			1	4												1				1				9
	Calliphoridae	saprovorous		organic matter	saprovorous						6	1	1		2	6		3			11			2	1				2		1	3		2						41
	Tachinidae	parasite		insects	parasite							1	1		1			2		1	12				1						1	1			1				1	23
	Ephydridae	herbivorous		plants	herbivorous								2			1											1							1						5
	Opomyzidae	herbivorous		plants	herbivorous										1																									1
	Trichoceridae	saprovorous		organic matter	saprovorous										1								2																	3
	Scatopsidae	saprovorous		organic matter	saprovorous										3	1																								4
	Agromyzidae	herbivorous		plants	herbivorous											2		1																		1				4
	Pipunculidae	parasite		insects	parasite													1																						1
	Stratiomyidae	saprovorous		organic matter	saprovorous																											1								1
COLEOPTERA																																								
	Atomaria	saprovorous		organic matter	saprovorous	1			2	1																		1			3	1								9
	Stilbus	saprovorous		organic matter	saprovorous	1						1											1						1											4
	Olibrus	saprovorous		organic matter	saprovorous	1															1																			2
	Ceutorhynchus assimilis	herbivorous		oil-seed rape	oil-seed rape	1	3			1	4					4	2	4	1	5	2	5		3	1	2	10	4	1		3	2	1	1						60
	Ceutorhynchus pallidactylus	herbivorous		oil-seed rape	oil-seed rape		1	1				1		6	4			4						1	1															19
	Ceutorhynchus melanostictus	herbivorous		oil-seed rape	oil-seed rape		1																																	1
	Ceutorhynchus rugulosus	herbivorous		oil-seed rape	oil-seed rape					1				2	1					3			1			1	2		1	1		1		1						15
	Ceutorhynchus	herbivorous		plants	herbivorous				1		1	1				1		1		2	2				1		1									1		1		13
	Curculionidae	herbivorous		plants	herbivorous	1																1	2								2							1		7
	Curculionidae inne	herbivorous		plants	herbivorous										1												1													2
	Phyllotreta	herbivorous		plants	herbivorous	2					1	1			1	2	1		1		1			2		1	1	1			4	1	1		1			1		23
	Chaetocnema	herbivorous		plants	herbivorous	1			1				4	1		3				1	3						2	2				2	2	2				3		27
	Longitarsus	herbivorous		plants	herbivorous	1	2						1			2			2	1	1				1	1	3	1	1		1		2		2				1	23
	Tachyporus	predator		insects	predator	5	3	6	4	3	3	3	12	6	4	15	7	2	4	14	5	5	1	5	4	4	8	3	2		8	2	5	5	10	3	3	3		167
	Philonthus	predator		insects	predator	1	4	3	1	15					2	4	1	2	1	12	1	1	2	2		1		2		2	2	1	3	1		3		2		69
	Gymnusa	predator		insects	predator	9		5	2	1	1	3			4	1	1		1	1			1	1		1	1	1			1					1	1			37
	Aleochara	saprovorous		organic matter	saprovorous	28	9	10	11	15	31	12	13	20	27	25	4	12	9	9	15	14	3	5	11	9	12	15	15	8	13	26	4	10	12	10	4	8	10	439
	Heterothops	predator		insects	predator	2		1			1					1			2		2	2				1					2		1					1		16
	Lathrobium	predator		insects	predator	3	1	3	7	2	1	1	4	4	4	7	1	3	6	3	5	2		2	2	2	2	3	1	1	2	1	2	1		2	3	1		82
	Oulema melanopus	herbivorous		crops	herbivorous		2	5	1	1	2				5	2	4	2		3	4	1		3	1	1	2	2	1	2	3	1			1	2				51
	Oulema cianella	herbivorous		crops	herbivorous																									1										1
	Cartodere	saprovorous		organic matter	saprovorous	1				1			1			1				1	1							1	1		1				1					10
	Meligethes	herbivorous		oil-seed rape	oil-seed rape	40	16	48	21	62	197	18	6	27	34	178	58	102	8	36	100	31	17	51	44	50	33	46	58	40	46	25	57	55	62	54	21	17	20	1678
	Aphodius	detrivorous		organic matter	saprovorous	3		1	1	1			2	1		7	2		2	5	1	1	3		2			1	1	1		2			1		1	1	1	41
	Cyphon	herbivorous		plants	herbivorous	2																1											1							4
	Bembidion	predator		insects	predator	1			1	1						4		1	1	1	3										2		1			1			1	18
	Amara	herbivorous		plants	herbivorous	2			1	1		4		2		4	3	4	1	2	6	2	1		3	4	5	1	1	2	9	2	2	3		7	2	1		75
	Poecilus	predator		insects	predator			1								1																	1		1	1				5
	Acrotrichis	saprovorous		organic matter	saprovorous			1					1		1															1										4
	Corticarina	saprovorous		organic matter	saprovorous				1		2				1	1	1	2	1										1	3	1	2								16
	Sitona	herbivorous		plants	herbivorous				1			1				1		1			1	1														1				7
	Oxytelus	saprovorous		organic matter	saprovorous					1	1				1			1		1	2					1		1		1	1		1					1	1	14
	Harmonia axyridis	predator		insects	predator					1																														1
	Agathidium	saprovorous		organic matter	saprovorous					2																														2
	Demetrias atricapillus	predator		insects	predator						1																													1
	Loricera	predator		insects	predator						3									1	1	1		1																7
	Quedius	predator		insects	predator						1																													1
	Thanatophilus rugosus	scavenger		carrion	saprovorous						1																										1			2
	Harpalus affinis	predator		insects	predator							1																												1
	Harpalus	predator		insects	predator									1						1	1					1	1	2												7
	Notoxus	saprovorous		organic matter	saprovorous							3							1						1						1					1		1		8
	Aphthona	herbivorous		plants	herbivorous								1						1																					2
	Phyllobius	herbivorous		plants	herbivorous								1																											1
	Haploglossa	saprovorous		organic matter	saprovorous									1																									1	2
	Glischrochilus	herbivorous		plants	herbivorous									1															1											2
	Glischrochilus quadrisignatus	herbivorous		plants	herbivorous																											1								1
	Agriotes	herbivorous		plants	herbivorous									1						6	1				1								1	1						11
	Corymbites	herbivorous		plants	herbivorous									1																										1
	Scymnus	predator		insects	predator										1							1		1							1									4
	Catops	saprovorous		organic matter	saprovorous											1									1						1									3
	Coccinella septempunctata	predator		insects	predator												1														1	1				1				4
	Cercyon	saprovorous		organic matter	saprovorous												1										1						1							3
	Onthophagus	saprovorous		organic matter	saprovorous														1							2														3
	Oedemera	herbivorous		plants	herbivorous														1																					1
	Cleonus	herbivorous		plants	herbivorous															1																				1
	Necrophorus	scavenger		carrion	saprovorous																1																			1
	Eumenidae	predator		insects	predator																	1																		1
	Acidota	saprovorous		organic matter	saprovorous																		1																	1
	Stenus	predator		insects	predator																		1																	1
	Trox	saprovorous		organic matter	saprovorous																		1																	1
	Trox sabulosus	saprovorous		organic matter	saprovorous																		1																	1
	Hister	predator		insects	predator																		2				2	1			1				1				1	8
	Elaphrus	predator		insects	predator																				1								1							2
	Elaphrus cupreus	predator		insects	predator																										2									2
	Cassida	herbivorous		plants	herbivorous																				1	1														2
	Aulacobaris lepidii	herbivorous		plants	herbivorous																							1												1
	Coccinellidae	predator		insects	predator																								1											1
	Propylea	herbivorous		plants	herbivorous																									1		1						1		3
	Clivina	predator		insects	predator																										1									1
	Calathus	predator		insects	predator																										1	1								2
	Psylliodes	herbivorous		plants	herbivorous																												1							1
	Anthicus	saprovorous		organic matter	saprovorous																													1						1
	Apion	herbivorous		plants	herbivorous																															1		1		2
	Chrysomela	herbivorous		plants	herbivorous																																1	1	1	3
	Coleoptera larva				herbivorous																																	4		4
HYMENOPTERA																																								
	Tenthredinidae		herbivorous	plants	herbivorous	1	4	1		1				1	2	1	7	2	1		1	1		1		2	1	2	1	4	9		1							44
	Tenthredo		herbivorous	plants	herbivorous	1					1																										1			3
	Dolerus		herbivorous	plants	herbivorous	2			1	1		1	1		3	1		1	1	3								3					1	1			1	1		22
	Apis mellifera		herbivorous	pollen	pollen		6	1				1														1		1	1					6		2				19
	Apidae		herbivorous	pollen	pollen	3	6	2		25	15			2	2	2	15	45	29	7	40	7	12	6	8	11	27	12	9	5	2	8	9	25		8	4		2	348
	Eulophidae		parasite	insects	parasite	1	1	1							1			1						1	1							3	1							11
	Diaeretus		parasite	insects	parasite	1							2	2		1								1	2	2		2						4	3	3		1	1	25
	Aphidius		parasite	aphids	parasite	3			1	1	2			1					1	1	3	1					1	5							1	2	2		1	26
	Formica		predator	insects	predator						1		1							1		1										1							1	6
	Formicidae		predator	insects	predator	1																										1								2
	Ichneumonidae		parasite	insects	parasite	2				2	1			4	2	3	1	2	1	1	2	1		1			3	3		2	1				4			1		37
	Monelata		parasite	insects	parasite	1		1		1	1		1					3	2		1						1				1		1							14
	Blacus		parasite	insects	parasite		1														1															1				3
	Habrocytus		parasite	insects	parasite		1																						1											2
	Pteromalidae		parasite	insects	parasite			1		1					1	1																								4
	Aphanogmus		parasite	insects	parasite				1												1	1					1				1									5
	Thersilochus		parasite	insects	parasite				1		1		1		1				1		1					1				3	2							1		13
	Asolcus		parasite	insects	parasite					1	3			1		1													1	1										8
	Bombus		herbivorous	pollen	pollen						3						6							1		3								1				1		15
	Rhizarcha		parasite	insects	parasite						1				1			1										2					1	1						7
	Trybliographa		parasite	insects	parasite						1				1					1			1						2	2	1	2	1						1	13
	Vespula		predator	insects	predator						1																			3		1								5
	Pompilidae		predator	insects	predator						6	1						2						1					1	1		1			1					14
	Xyella		herbivorous	plants	herbivorous							1																												1
	Platygaster		predator	insects	predator												1											1												2
	Nomada		herbivorous	pollen	pollen													3			1																			4
	Braconidae		parasite	insects	parasite														2																					2
	Myrmica		predator	insects	predator															1																				1
	Encyrtidae		parasite	insects	parasite																			1																1
	Lasius		predator	insects	predator																			1								1								2
	Harmolita		herbivorous	plants	herbivorous																				1															1
	Charips		herbivorous	plants	herbivorous																									1	1				5	1				8
	Polistes		predator	insects	predator																												1							1
HETEROPTERA																																								
	Dolycoris		herbivorous	plants	herbivorous	1																																		1
	Palomena		herbivorous	plants	herbivorous	1																				1				1										3
	Eremocoris erraticus		herbivorous	plants	herbivorous					1		1								4		1	1		1	1	2	1										1		14
	Aelia		herbivorous	plants	herbivorous					1					1																									2
	Lygus		herbivorous	plants	herbivorous						1								1	2													1							5
	Kleidocerys		herbivorous	plants	herbivorous							1	1		1					1		1	9			1		1	1	3		1			1					22
	Nabis		predator	insects	predator								1					1										1											1	4
	Pyrrhocoris		herbivorous	plants	herbivorous										1					1																				2
	Stenodema		herbivorous	plants	herbivorous										1																									1
	Thyreocoris		herbivorous	plants	herbivorous															1														1				1		3
	Thyreocoris scarabaeoides		herbivorous	plants	herbivorous											1																								1
	Eurydema		herbivorous	plants	herbivorous															1																				1
	Sehirus		herbivorous	plants	herbivorous																1																			1
	Chartoscirta		herbivorous	plants	herbivorous																	1																		1
	Orius		predator	insects	predator																		1																	1
	Saldidae		herbivorous	plants	herbivorous																						1													1
HOMOPTERA																																								
	Trioza		herbivorous	plants	herbivorous	1	2	1		1			1		2	1	4			1	3				2	2		1	1		1	1	2	1						28
	Calligypona		herbivorous	plants	herbivorous	1		1				1		1	2	7	2	1	3	1					2	2					1	3	3			4		1	2	38
	Empoasca		herbivorous	plants	herbivorous			3	1	1	1				1			2	1		2		3	1	1	1		1	1	1	2		1	3						27
	Aphalara		herbivorous	plants	herbivorous					1										1	2						1		1			1								7
	Trialeurodes		herbivorous	plants	herbivorous								1																											1
	Psammotettix		herbivorous	plants	herbivorous																1	1																		2
	Philaenus		herbivorous	plants	herbivorous																		2																	2
	Aphrodes		herbivorous	plants	herbivorous																				1															1
ORTHOPTERA																																								
	Tetrix		herbivorous	plants	herbivorous				1										1		1				1						1								1	6
	Tettigonidae larva		herbivorous	plants	herbivorous																						1													1
LEPIDOPTERA																																								
	Gonepteryx rhamni		herbivorous	plants	herbivorous					1																														1
	Pieris		herbivorous	plants	herbivorous												1																							1
	Eriocraniidae		herbivorous	plants	herbivorous																			1																1
	Crambus		herbivorous	plants	herbivorous							1																												1
NEUROPTERA																																								
	Raphidia notata		predator	insects	predator					1																														1
	Chrysopa		predator	insects	predator																			1														1		2
																																							
																																							
				Total per field		428	352	313	303	416	673	431	313	502	370	507	256	442	317	265	500	278	158	188	252	380	422	746	656	239	366	542	411	268	345	497	222	348	210

**Table 5:**
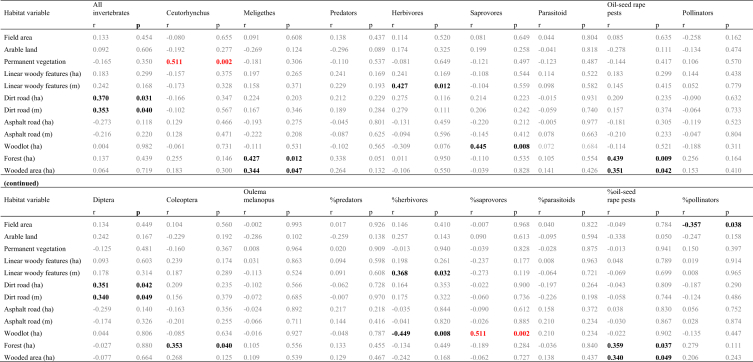
Pearson correlation coefficients testing the relationships between abundance and percentage (%) in the community of major invertebrate groups, and habitat variables of 34 oilseed rape fields. The relationships that meet the FDR-adjusted *P*-value are shown in red font; black font – *P* ≤ 0.05; grey font – *P* > 0.05.

**Table 6:** Results of *post-hoc* tests (Tukey's test with Spjotvoll and Stoline modification for an unequal sample size) comparing the concentrations of 12 chemical elements between the different groupings of organisms of oilseed rape crops depicted on Fig. 2 in Ref [1]; statistically significant differences (at *P* ≤ 0.05) are shown in bold.

K	Plant {1}	{2}	{3}	{4}	{5}	{6}	{7}	{8}	{9}	{10}	{11}	{12}	{13}	{14}
POLLINATORS {2}	1													
Ceutorhynchus spp. {3}	**0.000**	**0.000**												
Meligethes aeneus {4}	**0.000**	**0.000**	1.000											
Oulema melanopus {5}	**0.000**	**0.000**	1.000	1.000										
other herbivores {6}	0.585	0.627	0.329	0.325	0.327									
Diptera {7}	1.000	1.000	**0.000**	**0.000**	**0.000**	0.369								
Aphodius sp. {8}	**0.000**	**0.000**	1.000	1.000	1.000	0.217	**0.000**							
other saprophages {9}	**0.048**	0.056	1.000	1.000	1.000	0.987	**0.020**	0.999						
Coleoptera {10}	**0.000**	**0.000**	**0.000**	**0.000**	**0.000**	1.000	**0.000**	**0.000**	0.936					
Hister sp. {11}	**0.010**	**0.013**	0.643	0.639	0.641	1.000	**0.002**	0.464	1.000	0.984				
Coccinella septempunctata {12}	**0.000**	**0.000**	1.000	1.000	1.000	0.206	**0.000**	1.000	0.999	0.086	0.776			
other predators {13}	0.998	0.998	0.567	0.564	0.566	1.000	0.988	0.463	0.960	1.000	0.999	0.451		
PARASITES {14}	0.977	0.982	0.789	0.787	0.788	1.000	0.932	0.699	0.995	1.000	1.000	0.687	1.000	
All other insects {15}	**0.000**	**0.000**	**0.000**	**0.000**	**0.000**	1.000	0.000	**0.000**	0.998	0.904	1.000	0.370	1.000	1.000
Na	Plant {1}	{2}	{3}	{4}	{5}	{6}	{7}	{8}	{9}	{10}	{11}	{12}	{13}	{14}
POLLINATORS {2}	**0.000**													
Ceutorhynchus spp. {3}	0.172	**0.000**												
Meligethes aeneus {4}	**0.000**	0.837	**0.000**											
Oulema melanopus {5}	0.980	**0.000**	**0.004**	**0.000**										
other herbivores {6}	0.086	**0.000**	**0.002**	**0.000**	0.363									
Diptera {7}	**0.000**	0.987	**0.000**	1.000	**0.000**	**0.000**								
Aphodius sp. {8}	0.997	**0.000**	0.995	**0.000**	0.353	0.016	**0.000**							
other saprophages {9}	0.151	**0.000**	**0.008**	**0.000**	0.453	1.000	**0.000**	0.041						
Coleoptera {10}	**0.000**	0.735	**0.000**	**0.002**	**0.000**	**0.000**	**0.019**	**0.000**	**0.000**					
Hister sp. {11}	0.305	**0.000**	**0.006**	**0.000**	0.822	1.000	**0.000**	0.060	0.999	**0.000**				
Coccinella septempunctata {12}	**0.017**	1.000	0.337	1.000	**0.002**	**0.000**	1.000	0.091	**0.000**	1.000	**0.000**			
other predators {13}	0.341	**0.000**	**0.050**	**0.000**	0.653	1.000	**0.000**	0.150	1.000	**0.000**	0.999	**0.000**		
PARASITES {14}	0.817	**0.000**	0.293	**0.002**	0.970	1.000	**0.001**	0.565	1.000	**0.000**	1.000	**0.000**	1.000	
All other insects {15}	1.000	**0.000**	**0.008**	**0.000**	1.000	0.212	**0.000**	0.784	0.302	**0.000**	0.611	**0.004**	0.517	0.926
Ca	Plant {1}	{2}	{3}	{4}	{5}	{6}	{7}	{8}	{9}	{10}	{11}	{12}	{13}	{14}
POLLINATORS {2}	**0.000**													
Ceutorhynchus spp. {3}	**0.000**	0.706												
Meligethes aeneus {4}	**0.000**	**0.000**	**0.000**											
Oulema melanopus {5}	0.791	**0.000**	0.116	**0.000**										
other herbivores {6}	1.000	0.094	0.507	**0.000**	0.998									
Diptera {7}	**0.008**	**0.002**	0.740	**0.000**	0.995	0.945								
Aphodius sp. {8}	**0.001**	0.673	1.000	0.000	0.377	0.631	0.989							
other saprophages {9}	1.000	0.250	0.717	0.002	1.000	1.000	0.980	0.807						
Coleoptera {10}	**0.000**	**0.000**	**0.000**	1.000	**0.000**	**0.000**	**0.000**	**0.000**	**0.000**					
Hister sp. {11}	1.000	**0.007**	0.151	**0.000**	0.982	1.000	0.746	0.244	1.000	**0.000**				
Coccinella septempunctata {12}	**0.000**	0.378	0.056	1.000	**0.000**	**0.000**	**0.004**	**0.033**	**0.000**	1.000	**0.000**			
other predators {13}	1.000	0.950	0.999	0.218	1.000	1.000	1.000	1.000	1.000	0.121	1.000	**0.039**		
PARASITES {14}	1.000	0.459	0.838	**0.018**	0.999	1.000	0.988	0.893	1.000	**0.008**	1.000	**0.002**	1.000	
All other insects {15}	**0.000**	0.999	0.999	**0.000**	**0.005**	0.249	0.063	0.995	0.469	**0.000**	**0.038**	0.164	0.990	0.663
Mg	Plant {1}	{2}	{3}	{4}	{5}	{6}	{7}	{8}	{9}	{10}	{11}	{12}	{13}	{14}
POLLINATORS {2}	**0.000**													
Ceutorhynchus spp. {3}	**0.000**	**0.000**												
Meligethes aeneus {4}	**0.000**	**0.000**	**0.001**											
Oulema melanopus {5}	0.553	**0.000**	0.145	**0.000**										
other herbivores {6}	0.488	1.000	**0.000**	**0.000**	**0.050**									
Diptera {7}	**0.000**	**0.000**	**0.000**	**0.000**	**0.000**	0.657								
Aphodius sp. {8}	**0.000**	**0.000**	1.000	0.051	0.057	**0.000**	**0.000**							
other saprophages {9}	0.979	1.000	0.052	**0.000**	0.582	1.000	0.410	**0.030**						
Coleoptera {10}	**0.000**	**0.000**	1.000	**0.001**	0.060	0.000	**0.000**	1.000	0.036					
Hister sp. {11}	0.987	0.901	**0.004**	**0.000**	0.386	0.994	**0.002**	0.001	1.000	**0.002**				
Coccinella septempunctata {12}	**0.001**	**0.000**	0.708	1.000	0.053	**0.000**	**0.000**	0.830	**0.000**	0.790	**0.000**			
other predators {13}	0.773	1.000	0.026	**0.000**	0.292	1.000	0.996	**0.016**	1.000	**0.019**	0.996	**0.000**		
PARASITES {14}	0.993	1.000	0.215	**0.005**	0.806	1.000	0.814	0.154	1.000	0.174	1.000	**0.001**	1.000	
All other insects {15}	**0.000**	0.455	**0.000**	**0.000**	**0.000**	0.999	0.059	**0.000**	0.968	**0.000**	0.202	**0.000**	1.000	0.998
Cu	Plant {1}	{2}	{3}	{4}	{5}	{6}	{7}	{8}	{9}	{10}	{11}	{12}	{13}	{14}
POLLINATORS {2}	0.983													
Ceutorhynchus spp. {3}	**0.000**	**0.000**												
Meligethes aeneus {4}	**0.000**	**0.000**	**0.001**											
Oulema melanopus {5}	**0.000**	**0.000**	1.000	**0.000**										
other herbivores {6}	0.999	1.000	**0.008**	0.588	**0.002**									
Diptera {7}	**0.000**	**0.000**	0.225	**0.000**	0.954	**0.000**								
Aphodius sp. {8}	**0.000**	**0.000**	1.000	0.076	0.991	**0.018**	0.190							
other saprophages {9}	1.000	1.000	**0.006**	0.377	**0.002**	1.000	**0.000**	**0.013**						
Coleoptera {10}	**0.000**	**0.000**	**0.000**	**0.000**	**0.000**	**0.000**	**0.000**	**0.000**	**0.028**					
Hister sp. {11}	1.000	1.000	**0.000**	**0.003**	**0.000**	0.999	**0.000**	**0.000**	1.000	**0.001**				
Coccinella septempunctata {12}	0.486	0.845	0.383	1.000	0.179	0.992	**0.025**	0.546	0.918	**0.000**	0.488			
other predators {13}	**0.001**	**0.006**	1.000	0.831	1.000	**0.028**	1.000	1.000	**0.005**	**0.000**	**0.001**	0.349		
PARASITES {14}	1.000	1.000	**0.029**	0.500	**0.012**	1.000	**0.002**	**0.049**	1.000	0.362	1.000	0.923	**0.001**	
All other insects {15}	**0.001**	**0.000**	**0.000**	**0.000**	**0.000**	0.355	**0.000**	**0.000**	0.940	**0.000**	0.873	**0.007**	**0.000**	0.999
Zn	Plant {1}	{2}	{3}	{4}	{5}	{6}	{7}	{8}	{9}	{10}	{11}	{12}	{13}	{14}
POLLINATORS {2}	1.000													
Ceutorhynchus spp. {3}	**0.000**	**0.000**												
Meligethes aeneus {4}	0.856	0.998	**0.001**											
Oulema melanopus {5}	**0.000**	**0.001**	1.000	0.057										
other herbivores {6}	**0.000**	**0.000**	**0.000**	**0.000**	**0.000**									
Diptera {7}	**0.000**	**0.000**	**0.000**	**0.000**	**0.000**	0.535								
Aphodius sp. {8}	**0.000**	**0.000**	1.000	**0.008**	1.000	**0.000**	**0.000**							
other saprophages {9}	0.900	0.802	**0.014**	0.546	**0.030**	0.381	1.000	**0.012**						
Coleoptera {10}	0.999	1.000	**0.000**	1.000	**0.005**	**0.000**	**0.000**	**0.001**	0.726					
Hister sp. {11}	1.000	1.000	0.371	1.000	0.602	**0.000**	**0.038**	0.340	0.839	1.000				
Coccinella septempunctata {12}	0.715	0.853	1.000	0.979	1.000	**0.000**	**0.000**	1.000	**0.022**	0.913	0.812			
other predators {13}	**0.000**	**0.000**	**0.000**	**0.000**	**0.000**	**0.038**	**0.000**	**0.000**	**0.000**	**0.000**	**0.000**	**0.000**		
PARASITES {14}	0.926	0.860	0.060	0.676	0.102	0.877	1.000	0.055	1.000	0.807	0.885	0.081	**0.000**	
All other insects {15}	**0.001**	**0.019**	0.792	0.351	0.998	**0.000**	**0.000**	0.905	0.106	**0.041**	0.930	1.000	**0.000**	0.234
Fe	Plant {1}	{2}	{3}	{4}	{5}	{6}	{7}	{8}	{9}	{10}	{11}	{12}	{13}	{14}
POLLINATORS {2}	**0.000**													
Ceutorhynchus spp. {3}	0.293	**0.000**												
Meligethes aeneus {4}	**0.000**	**0.000**	0.594											
Oulema melanopus {5}	0.756	**0.000**	1.000	0.558										
other herbivores {6}	0.330	1.000	**0.022**	**0.001**	**0.035**									
Diptera {7}	**0.000**	1.000	**0.000**	**0.000**	**0.000**	1.000								
Aphodius sp. {8}	0.180	**0.000**	1.000	0.994	1.000	**0.008**	**0.000**							
other saprophages {9}	0.998	0.989	0.789	0.286	0.853	0.999	0.971	0.624						
Coleoptera {10}	**0.000**	**0.000**	**0.001**	0.547	**0.001**	**0.000**	**0.000**	0.093	**0.044**					
Hister sp. {11}	1.000	**0.003**	1.000	0.948	1.000	0.113	**0.001**	1.000	0.966	0.344				
Coccinella septempunctata {12}	0.984	**0.001**	1.000	1.000	1.000	**0.004**	**0.001**	1.000	0.505	0.998	1.000			
other predators {13}	0.095	0.999	**0.009**	**0.001**	**0.013**	0.994	1.000	**0.004**	0.649	**0.000**	**0.035**	**0.003**		
PARASITES {14}	0.998	1.000	0.866	0.471	0.906	1.000	1.000	0.755	1.000	0.142	0.976	0.666	0.803	
All other insects {15}	**0.000**	**0.000**	**0.000**	**0.045**	**0.000**	**0.000**	**0.000**	**0.005**	**0.015**	0.999	0.123	0.970	**0.000**	0.069
Mn	Plant {1}	{2}	{3}	{4}	{5}	{6}	{7}	{8}	{9}	{10}	{11}	{12}	{13}	{14}
POLLINATORS {2}	**0.042**													
Ceutorhynchus spp. {3}	0.931	**0.000**												
Meligethes aeneus {4}	0.969	**0.000**	1.000											
Oulema melanopus {5}	1.000	**0.021**	1.000	1.000										
other herbivores {6}	**0.001**	0.117	**0.000**	**0.000**	**0.000**									
Diptera {7}	0.094	1.000	**0.000**	**0.000**	0.068	0.077								
Aphodius sp. {8}	0.876	**0.000**	1.000	1.000	0.996	**0.000**	**0.001**							
other saprophages {9}	1.000	0.924	1.000	1.000	1.000	**0.001**	0.959	1.000						
Coleoptera {10}	1.000	0.060	0.889	0.942	1.000	**0.002**	0.131	0.828	1.000					
Hister sp. {11}	1.000	0.942	1.000	1.000	1.000	**0.001**	0.978	1.000	1.000	1.000				
Coccinella septempunctata {12}	1.000	1.000	0.996	0.998	1.000	**0.017**	1.000	0.985	0.997	1.000	1.000			
other predators {13}	0.177	0.757	0.052	0.063	0.111	1.000	0.686	**0.035**	**0.038**	0.190	0.141	0.447		
PARASITES {14}	1.000	0.998	1.000	1.000	1.000	0.057	0.999	1.000	1.000	1.000	1.000	1.000	0.083	
All other insects {15}	1.000	**0.025**	0.968	0.988	1.000	**0.001**	0.059	0.926	1.000	1.000	1.000	1.000	0.161	1.000
As	Plant {1}	{2}	{3}	{4}	{5}	{6}	{7}	{8}	{9}	{10}	{11}	{12}	{13}	{14}
POLLINATORS {2}	1.000													
Ceutorhynchus spp. {3}	1.000	1.000												
Meligethes aeneus {4}	1.000	1.000	1.000											
Oulema melanopus {5}	1.000	1.000	1.000	1.000										
other herbivores {6}	1.000	1.000	1.000	1.000	1.000									
Diptera {7}	0.995	1.000	0.996	0.996	0.999	1.000								
Aphodius sp. {8}	1.000	1.000	1.000	1.000	1.000	1.000	0.999							
other saprophages {9}	1.000	1.000	1.000	1.000	1.000	1.000	1.000	1.000						
Coleoptera {10}	0.915	0.990	0.932	0.917	0.976	1.000	1.000	0.984	1.000					
Hister sp. {11}	1.000	1.000	1.000	1.000	1.000	1.000	1.000	1.000	1.000	1.000				
Coccinella septempunctata {12}	1.000	1.000	1.000	1.000	1.000	1.000	1.000	1.000	1.000	1.000	1.000			
other predators {13}	1.000	1.000	1.000	1.000	1.000	1.000	1.000	1.000	1.000	1.000	1.000	1.000		
PARASITES {14}	1.000	1.000	1.000	1.000	1.000	1.000	1.000	1.000	1.000	1.000	1.000	1.000	1.000	
All other insects {15}	**0.000**	**0.000**	**0.000**	**0.000**	**0.000**	**0.018**	**0.000**	**0.000**	0.088	**0.000**	**0.001**	**0.018**	0.359	0.355
Cd	Plant {1}	{2}	{3}	{4}	{5}	{6}	{7}	{8}	{9}	{10}	{11}	{12}	{13}	{14}
POLLINATORS {2}	**0.035**													
Ceutorhynchus spp. {3}	**0.005**	**0.000**												
Meligethes aeneus {4}	0.602	**0.000**	0.886											
Oulema melanopus {5}	0.069	**0.000**	1.000	0.990										
other herbivores {6}	0.908	0.141	1.000	1.000	1.000									
Diptera {7}	0.846	**0.000**	0.676	1.000	0.941	0.999								
Aphodius sp. {8}	0.118	**0.000**	1.000	0.995	1.000	1.000	0.967							
other saprophages {9}	1.000	1.000	0.992	1.000	0.996	0.954	1.000	0.997						
Coleoptera {10}	**0.003**	1.000	**0.000**	**0.000**	**0.000**	0.087	**0.000**	**0.000**	0.998					
Hister sp. {11}	1.000	0.997	0.746	0.995	0.821	0.776	0.999	0.834	1.000	0.985				
Coccinella septempunctata {12}	1.000	0.726	1.000	1.000	1.000	1.000	1.000	1.000	1.000	0.600	0.999			
other predators {13}	1.000	1.000	1.000	1.000	1.000	0.996	1.000	1.000	1.000	1.000	1.000	1.000		
PARASITES {14}	1.000	1.000	0.999	1.000	0.999	0.990	1.000	0.999	1.000	1.000	1.000	1.000	1.000	
All other insects {15}	0.065	1.000	**0.000**	**0.000**	**0.000**	0.191	**0.000**	**0.000**	1.000	1.000	0.999	0.803	1.000	1.000
Co	Plant {1}	{2}	{3}	{4}	{5}	{6}	{7}	{8}	{9}	{10}	{11}	{12}	{13}	{14}
POLLINATORS {2}	**0.000**													
Ceutorhynchus spp. {3}	**0.014**	**0.000**												
Meligethes aeneus {4}	1.000	**0.000**	0.026											
Oulema melanopus {5}	0.542	**0.000**	1.000	0.659										
other herbivores {6}	1.000	**0.000**	1.000	1.000	1.000									
Diptera {7}	0.000	**0.003**	**0.000**	**0.000**	**0.000**	**0.000**								
Aphodius sp. {8}	0.599	**0.000**	1.000	0.708	1.000	1.000	**0.000**							
other saprophages {9}	1.000	**0.000**	1.000	1.000	1.000	1.000	**0.000**	1.000						
Coleoptera {10}	0.000	**0.000**	**0.000**	**0.000**	**0.000**	**0.000**	**0.000**	**0.000**	**0.000**					
Hister sp. {11}	1.000	**0.000**	0.987	1.000	1.000	1.000	**0.000**	1.000	1.000	**0.000**				
Coccinella septempunctata {12}	1.000	**0.000**	1.000	1.000	1.000	1.000	**0.000**	1.000	1.000	**0.000**	1.000			
other predators {13}	1.000	**0.000**	1.000	1.000	1.000	1.000	**0.000**	1.000	1.000	**0.000**	1.000	1.000		
PARASITES {14}	1.000	**0.000**	1.000	1.000	1.000	1.000	**0.000**	1.000	1.000	**0.000**	1.000	1.000	1.000	
All other insects {15}	**0.000**	**0.000**	**0.000**	**0.000**	**0.000**	0.162	**0.000**	**0.000**	0.282	**0.000**	**0.001**	0.079	0.703	0.582
Pb	Plant {1}	{2}	{3}	{4}	{5}	{6}	{7}	{8}	{9}	{10}	{11}	{12}	{13}	{14}
POLLINATORS {2}	**0.000**													
Ceutorhynchus spp. {3}	**0.000**	0.239												
Meligethes aeneus {4}	**0.000**	**0.002**	0.986											
Oulema melanopus {5}	**0.000**	**0.019**	0.997	1.000										
other herbivores {6}	1.000	**0.000**	**0.006**	**0.039**	**0.036**									
Diptera {7}	**0.000**	**0.038**	1.000	1.000	1.000	**0.015**								
Aphodius sp. {8}	**0.000**	0.068	1.000	1.000	1.000	**0.026**	1.000							
other saprophages {9}	0.970	**0.000**	**0.000**	**0.003**	**0.003**	0.998	**0.001**	**0.002**						
Coleoptera {10}	**0.000**	0.913	0.999	0.377	0.716	**0.001**	0.899	0.896	**0.000**					
Hister sp. {11}	1.000	**0.000**	**0.000**	**0.002**	**0.002**	1.000	**0.000**	**0.001**	0.998	**0.000**				
Coccinella septempunctata {12}	**0.009**	1.000	1.000	1.000	1.000	**0.001**	1.000	1.000	**0.000**	1.000	**0.001**			
other predators {13}	1.000	**0.006**	0.068	0.182	0.176	1.000	0.111	0.148	1.000	**0.029**	1.000	**0.030**		
PARASITES {14}	0.976	**0.000**	**0.006**	**0.022**	**0.021**	0.998	**0.011**	**0.016**	1.000	**0.002**	0.998	**0.002**	1.000	
All other insects {15}	**0.000**	**0.000**	**0.000**	**0.000**	**0.000**	**0.000**	**0.000**	**0.000**	**0.000**	**0.000**	**0.000**	0.052	**0.000**	**0.000**

**Table 7:**
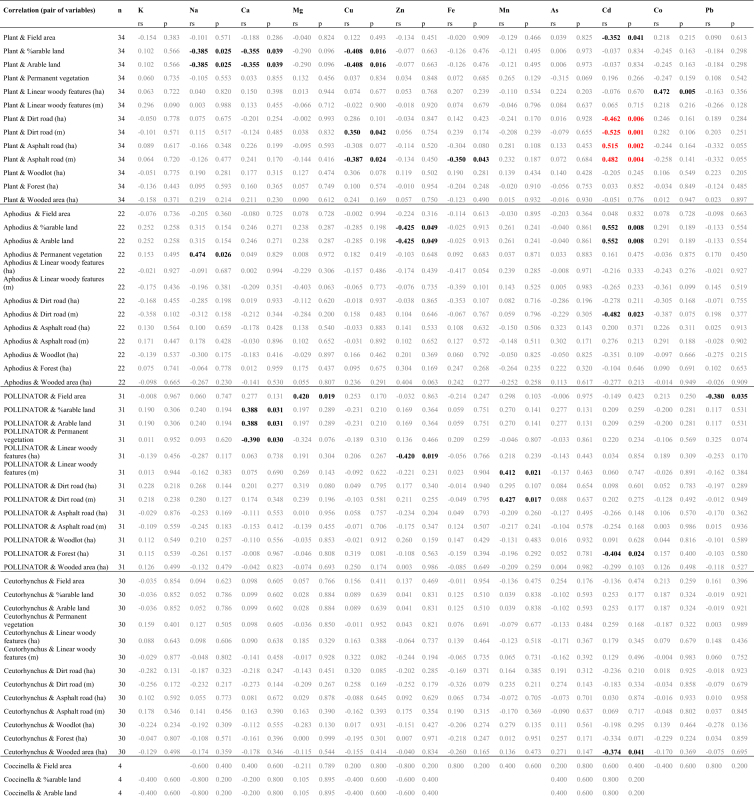

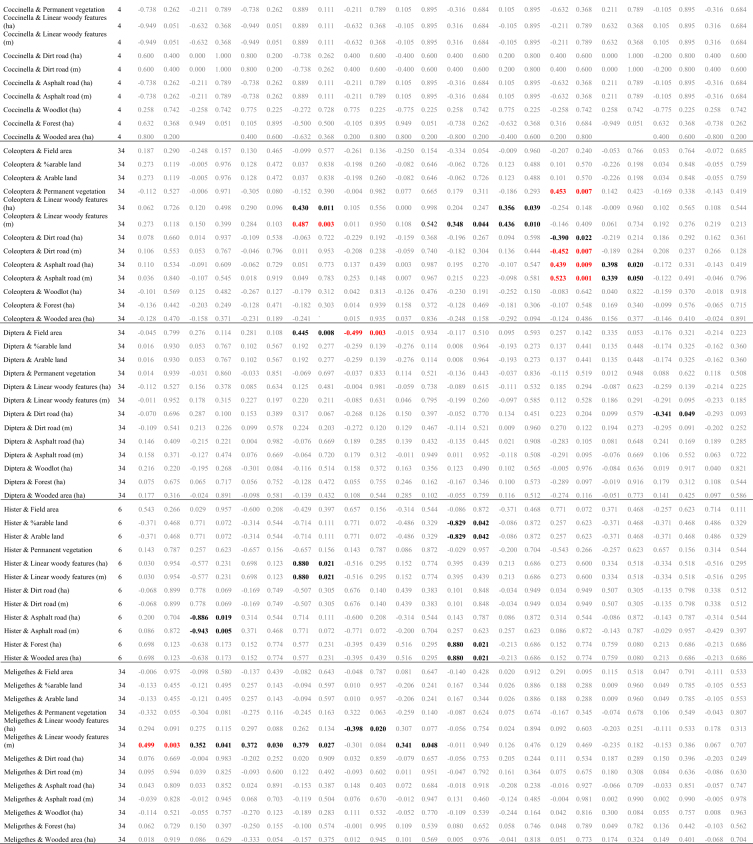

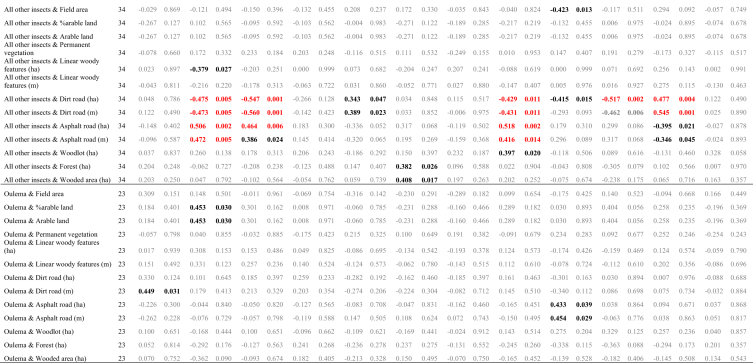
Results of Spearman rank correlations (r_s_) and associated *p*-values testing the relationship between the concentrations of 12 elements measured in oilseed rape plant samples (determined as the whole plant) and major functional invertebrate groupings or main taxonomic insect groups, and habitat variables of 34 oilseed rape fields in SW Poland in spring 2015. The relationships that meet the FDR-adjusted *P*-value are shown in red font; black font – *P* ≤ 0.05; grey font – *P* > 0.05.

The most numerous of the invertebrates, classified into six functional groups, were herbivores, which made up on average 39.4% (95% CI = 34.9–43.9%) of all the specimens sampled from one field. Next in order of abundance were saprophages, 36.5% (31.7–41.3%); pests of oilseed rape, 13.7% (10.9–16.6%); predators, 5.7% (4.4–7.0%); pollinators (wild bees, primarily representatives of two sub-families, Andreninae and Halictinae), 3.2% (2.0–4.3%); and parasitoids, 1.5% (1.2–1.8%) (Annex 1 in Ref [1]). 18% fewer invertebrates were sampled on small fields with mosaic-like surroundings compared with small fields with open surroundings (Fig. 1 in Ref [1]).

### 1.1 Relationships between landscape and habitat variables, and abundance of invertebrates

There was a weak but insignificant relationship between the (*log*-transformed) area of a field and the number of invertebrates sampled there (*r* = 0.155, *P* = 0.406). On average, 6.2% more invertebrates were sampled in the large fields (i.e. area >13 ha; *n* = 24) than in the small ones (i.e. area <8 ha; *n* = 10): 387 (95% C.I. = 334–440) vs. 363 (237–488), respectively. More pronounced differences emerged when the landscape context, i.e. the type of field surroundings, was taken into account. In an open landscape, 12% fewer invertebrates were sampled in the small fields than in the large ones (Fig. 1 in Ref [1]). In a mosaic-like landscape, by contrast, 20% more invertebrates were sampled in the large fields than in the small ones (Fig. 1 in Ref [1]). 18% fewer invertebrates were sampled in small fields with mosaic surroundings than in small fields with open surroundings (Fig. 1 in Ref [1]). None of these differences, however, were statistically significant (Mann-Whitney test, *P* ≥ 0.201), presumably because of the small sample size and the confounding influence of edge habitat variability (see below).

Analysis of the eight edge habitat variables measured within a 100 m radius of the invertebrate sampling points (Table 1) in the 34 oilseed rape fields (Fig. 1) with respect to the number or percentage (%) of six functional invertebrate groupings and the most numerous insect orders sampled there yielded only a few statistically significant relationships. But the *P*-value of none of them met the threshold for multiple comparisons (at *P* ≤ 0.0043; *k* = 12). Specifically, we found that the number of all invertebrates sampled was positively correlated with the area of dirt roads (Pearson correlation coefficient, *r* = 0.307, *P* = 0.031). Further, both the number and percentage (%) of the herbivorous insects were positively correlated with the length of linear woody features surrounding the fields (*r* = 0.427 and 0.368, *P* = 0.012 and 0.032, respectively; Fig. 2A); the number of saprophages was positively correlated with the area of woods (*r* = 0.447, *P* = 0.008; Fig. 2B); the number and percentage (%) of oilseed rape pests were positively correlated with the forest (*r* = 0.439 and 0.359, *P* = 0.009 and 0.037, respectively) and wooded area (*r* = 0.359 and 0.344, *P* = 0.037 and 0.046, respectively); and the numbers of all invertebrates and Diptera (all species) were correlated with the length of dirt roads (*r* = 0.363 and 0.340, *P* = 0.045 and 0.049, respectively). Interestingly, we found that the number of the pooled four species of true weevils of the tribe *Ceutorhynchus* spp. (*C. assimilis = C. obstrictus*, *C. pallidactylus*, *C. melanostictus*, *C. rugulosus*) was positively correlated with the area of permanent vegetation (*r* = 0.512, *P* = 0.002; Fig. 2C), while the number of pollen beetles *Meligethes aeneus* was positively correlated with the area of forests (*r* = 0.427, *P* = 0.012; Fig. 2D), woods (*r* = 0.351 and, *P* = 0.042), and with the total wooded area (*r* = 0.344, *P* = 0.047). Similarly, the number of Coleoptera (all species) was positively correlated with the area of forests (*r* = 0.353, *P* = 0.040).

The only negative statistically significant relationship was between the percentage (%) of herbivorous insects and the area of woods (*r* = −0.462, *P* = 0.009).

The concentrations of Na, Ca, Mg, Cu, Zn, Fe, As, Co and Pb varied significantly between two oilseed rape pest taxa, *M. aeneus* and *Ceutorhynchus* spp., sampled on the same fields (*t*-test for dependent samples, in all cases, *P* ≤ 0.008).

## 2 Experimental design, materials and method

### 2.1 The study area

The 35 winter oilseed rape fields were managed using conventional amounts of agrochemicals, including pesticides and fertilisers. The landowner (Top Farms Wielkopolska Co., Poland) supplied management data on agricultural practices in a few large fields for the study year; both the timing and use of agrochemicals were similar in all the other fields. The fields were sown (winter oilseed rape cultivar: PRW 31 F-1) in the second half of September 2014. Each of three mineral fertilisers (Polifoska G, Saletrosan 26% N, ammonium sulphate 34% N) was used in a dose of 300 kg ha^−1^; foliar fertiliser (OSD Bor, 1.5 kg ha^−1^) and magnesium sulphate (3 kg ha^−1^). Also applied were herbicides (Butisan Star Max; 2.5 L ha^−1^); insecticides between March and May (Ammo Super, Decis, Alfacet, Mospilan; each 0.1–0.15 L ha^−1^) targeting herbivorous insects, mostly stem weevils *Ceutorhynchus* spp. and pollen beetles *Meligethes* spp.; and fungicides (Caryx, 0.6 L ha^−1^; Pictor; 0.5 L ha^−1^).

### 2.2 Chemical analysis

We used reference materials for each of the AAS measurements. These were blind tests, i.e. they contained the same chemical composition as a particular sample, but were devoid of the analysed biological material. The analytical procedure for preparing these samples was the same as in the case of our ones. We used standardised samples obtained from SGAB Analytica, Luleå Technical University, Luleå, Sweden and Fürst Medical Laboratory, Billingstad, Oslo, Norway, Certified Values and Uncertainty NCS ZC, i.e. standards of a particular quality for each kind of tissue and chemical element. The analytical measurement process was validated using reference materials, i.e. CVU (bovine liver, kidney, muscles, lung, bone) provided by SGAB Analytica, Luleå Technical University, Luleå, Sweden and Fürst Medical Laboratory, Billingstad, Oslo, Norway, Certified Values and Uncertainty NCS ZC. Reference values amounted to 0.25 ± 0.05–32.7 ± 1.8 for the target chemical elements. The average (±SD) values determined for the target elements (20 measurements in the invertebrate and plant samples) were 0.19 ± 0.05–34.8 ± 1.07. The precision of the method, understood as the degree of conformity between the results of multiple analyses performed on the same sample, was 5% (relative standard deviation, RSD).

### 2.3 Data treatment

Pre-analysis of our habitat variables quantified for the individual oilseed rape fields showed strong collinear associations (tested by Spearman's and Pearson's correlation coefficients) between some of these variables. Principal Component Analysis (PCA) was applied to reduce collinearity among them (see Table 2). But because PCA outputs identified PC-axes that clustered structurally distinct variables (e.g. dirt and tarred roads or coverage of arable land and forest; Table 2), for which we wanted to assess their individual influence on particular invertebrate groups, the PCA-derived variables were of limited use in our subsequent analyses. Further, because the habitat variables were generally only loosely related to abundance data and the elemental traits of the studied organisms (and only single such relationships met the threshold of statistical significance), we assumed that the results of a univariate analysis (with *P*-values adjusted to multiple comparisons) would be justified, thus permitting a robust biological interpretation of our observations.

## Conflict of interest

The authors declare that they have no known competing financial interests or personal relationships that could have appeared to influence the work reported in this paper.
